# 中国慢性淋巴细胞白血病/小淋巴细胞淋巴瘤诊疗现状：一项多中心问卷调查研究

**DOI:** 10.3760/cma.j.issn.0253-2727.2023.05.005

**Published:** 2023-05

**Authors:** 卫 徐, 树华 易, 茹 冯, 欣 王, 洁 金, 坚青 糜, 凯阳 丁, 威 杨, 挺 牛, 少元 王, 可树 周, 宏凌 彭, 亮 黄, 丽宏 刘, 军 马, 军 罗, 丽萍 苏, 鸥 白, 林 刘, 菲 李, 鹏程 贺, 云 曾, 大 高, 明 江, 季石 王, 红霞 姚, 录贵 邱, 建勇 李

**Affiliations:** 1 江苏省人民医院（南京医科大学第一附属医院），南京 210029 Department of Hematology, the First Affiliated Hospital of Nanjing Medical University, Jiangsu Province Hospital, Nanjing 210029, China; 2 中国医学科学院血液病医院（中国医学科学院血液学研究所），实验血液学国家重点实验室，国家血液系统疾病临床医学研究中心，细胞生态海河实验室，天津 300020 State Key Laboratory of Experimental Hematology, National Clinical Research Center for Blood Diseases, Haihe Laboratory of Cell Ecosystem, Institute of Hematology & Blood Diseases Hospital, Chinese Academy of Medical Sciences & Peking Union Medical College, Tianjin 300020, China; 3 南方医科大学南方医院，广州 510515 Nanfang Hospital, Southern Medical University, Guangzhou 510515, China; 4 山东省立医院，济南 250021 Shandong Provincial Hospital, Jinan 250021, China; 5 浙江大学医学院附属第一医院，杭州 310003 The First Affiliated Hospital of Medical College of Zhejiang University, Hangzhou 310003, China; 6 上海交通大学医学院附属瑞金医院，上海 200025 Ruijin Hospital Affiliated to Medical College of Shanghai Jiaotong University, Shanghai 200025, China; 7 中国科学技术大学附属第一医院，合肥 230031 Anhui Province Cancer Hospital, Hefei 230031, China; 8 中国医科大学附属盛京医院，沈阳 117004 Shengjing Hospital Affiliated to China Medical University, Shenyang 117004, China; 9 四川大学华西医院，成都 610044 West China Hospital of Sichuan University, Chengdu 610044, China; 10 福建医科大学附属协和医院，福州 350001 Union Hospital Affiliated to Fujian Medical University, Fuzhou 350001, China; 11 河南省肿瘤医院（郑州大学附属肿瘤医院），郑州 450003 Henan Cancer Hospital（Affiliated Cancer Hospital of Zhengzhou University）, Zhengzhou 450003, China; 12 中南大学湘雅二医院，长沙 410008 Xiangya Second Hospital of Central South University, Changsha 410008, China; 13 华中科技大学同济医学院附属同济医院，武汉 430030 Tongji Hospital, Tongji Medical College, Huazhong University of Science and Technology, Wuhan 430030, China; 14 河北医科大学第四医院（河北省肿瘤医院），石家庄 050011 The Fourth Hospital of Hebei Medical University（Hebei Tumor Hospital）, Shijiazhuang 050011, China; 15 哈尔滨血液肿瘤研究所，哈尔滨 150001 Harbin Institute of hematological oncology, Harbin 150001, China; 16 广西医科大学第一附属医院，南宁 530021 The First Affiliated Hospital of Guangxi Medical University, Nanchang 530021, China; 17 山西省肿瘤医院，太原 030013 Shanxi Cancer Hospital, Taiyuan 030013, China; 18 吉林大学第一医院，长春 130061 The first hospital of Jilin University, Changchun 130061, China; 19 重庆医科大学附属第一医院，重庆 400042 The First Affiliated Hospital of Chongqing Medical University, Chongqing 400042, China; 20 南昌大学第一附属医院，南昌 330006 The First Affiliated Hospital of Nanchang University, Nanchang 330006, China; 21 西安交通大学第一附属医院，西安 710061 The First Affiliated Hospital of Xi' an Jiaotong University, Xi' an 710061, China; 22 昆明医科大学第一附属医院，昆明 650032 The First Affiliated Hospital of Kunming Medical University, Kunming 650032, China; 23 内蒙古医科大学附属医院，呼和浩特 750306 Affiliated Hospital of Inner Mongolia Medical University, Hohhot 750306, China; 24 新疆医科大学第一附属医院，乌鲁木齐 830011 The First Affiliated Hospital of Xinjiang Medical University, Urumqi 830011, China; 25 贵州医科大学附属医院，贵阳 550004 Affiliated hospital of Guizhou Medical University, Guiyang 550004, China; 26 海南省人民医院，海口 570311 Hainan Provincial People's Hospital, Haikou 570311, China

**Keywords:** 慢性淋巴细胞白血病, 问卷调查研究, 诊断, 治疗, Chronic lymphocytic leukemia, Survey research, Diagnosis, Therapeutic

## Abstract

**目的:**

了解慢性淋巴细胞白血病（CLL）/小淋巴细胞淋巴瘤（SLL）在中国各级医疗机构血液科、肿瘤科与淋巴瘤科的诊疗现状。

**方法:**

2021年3月至2021年7月期间国内多家医疗单位开展了一项多中心问卷调查，共纳入1000名符合研究条件的医生，采用面对面和线上问卷调查相结合的方式，利用标准化问卷对其所诊疗的CLL/SLL患者的疾病诊断、预后评估、治疗方案选择、布鲁顿酪氨酸激酶（BTK）抑制剂使用等方面进行调查。

**结果:**

①受访医生接诊的CLL/SLL患者男性比例高于女性（62.0％对38.0％），年龄主要集中在61～70岁（占37.7％）；②在患者诊断上，除血常规外，受访医生大多进行了骨髓穿刺涂片、活检、免疫组化等检测；③仅13.7％的受访医生完全掌握了现有指南推荐的起始治疗指征；④在预后高危因素认知方面，受访医生对伴免疫球蛋白重链可变区（IGHV）未突变、11q−的掌握远不如TP53突变和复杂核型这两个预后高危因素，且仅有17.1％的受访医生完全掌握了CLL国际预后指数（CLL-IPI）评分系统；⑤在一线治疗方案中，BTK抑制剂是不同类型患者的一线治疗方案，且医生对伴高危因素和老年患者需优先使用BTK抑制剂已形成一定认知，但不同类型患者实际使用BTK抑制剂比例不高（31.6％～46.0％）；⑥69.0％的受访医生曾减量使用过BTK抑制剂，66.8％的医生曾中断BTK抑制剂超过12 d。导致减量或中断BTK抑制剂使用的主要原因为不良反应（房颤、重度骨髓抑制、出血、肺部感染等）、经济因素以及病情得到控制；⑦血液科医生和肿瘤科医生在CLL/SLL预后评估、治疗方案选择、BTK抑制剂应用等方面均存在一定差异。

**结论:**

目前中国各级医院血液科、肿瘤科与淋巴瘤科医生在CLL/SLL的诊断、治疗指征判定、预后评估、伴随疾病评估、治疗方案选择以及BTK抑制剂使用上仍有很多不足，由不良反应导致减量或中断/终止治疗的患者占比较高。

## Abstract (en)

**Objective:**

To understand the current status of diagnosis and treatment of chronic lymphocytic leukemia（CLL）/small lymphocytic lymphoma（SLL）among hematologists, oncologists, and lymphoma physicians from hospitals of different levels in China.

**Methods:**

This multicenter questionnaire survey was conducted from March 2021 to July 2021 and included 1,000 eligible physicians. A combination of face-to-face interviews and online questionnaire surveys was used. A standardized questionnaire regarding the composition of patients treated for CLL/SLL, disease diagnosis and prognosis evaluation, concomitant diseases, organ function evaluation, treatment selection, and Bruton tyrosine kinase（BTK）inhibitor was used.

**Results:**

①The interviewed physicians stated that the proportion of male patients treated for CLL/SLL is higher than that of females, and the age is mainly concentrated in 61–70 years old. ②Most of the interviewed physicians conducted tests, such as bone marrow biopsies and immunohistochemistry, for patient diagnosis, in addition to the blood test. ③Only 13.7％ of the interviewed physicians fully grasped the initial treatment indications recommended by the existing guidelines. ④In terms of cognition of high-risk prognostic factors, physicians' knowledge of unmutated immunoglobulin heavy-chain variable and 11q- is far inferior to that of TP53 mutation and complex karyotype, which are two high-risk prognostic factors, and only 17.1％ of the interviewed physicians fully mastered CLL International Prognostic Index scoring system. ⑤Among the first-line treatment strategy, BTK inhibitors are used for different types of patients, and physicians have formed a certain understanding that BTK inhibitors should be preferentially used in patients with high-risk factors and elderly patients, but the actual use of BTK inhibitors in different types of patients is not high（31.6％–46.0％）. ⑥BTK inhibitors at a reduced dose in actual clinical treatment were used by 69.0％ of the physicians, and 66.8％ of the physicians had interrupted the BTK inhibitor for >12 days in actual clinical treatment. The use of BTK inhibitors is reduced or interrupted mainly because of adverse reactions, such as atrial fibrillation, severe bone marrow suppression, hemorrhage, and pulmonary infection, as well as patients' payment capacity and effective disease progression control. ⑦Some differences were found in the perceptions and behaviors of hematologists and oncologists regarding the prognostic assessment of CLL/SLL, the choice of treatment options, the clinical use of BTK inhibitors, etc.

**Conclusion:**

At present, a gap remains between the diagnosis and treatment of CLL/SLL among Chinese physicians compared with the recommendations in the guidelines regarding the diagnostic criteria, treatment indications, prognosis assessment, accompanying disease assessment, treatment strategy selection, and rational BTK inhibitor use, especially the proportion of dose reduction or BTK inhibitor discontinuation due to high adverse events.

慢性淋巴细胞白血病（CLL）/小淋巴细胞淋巴瘤（SLL）是主要发生在中老年人群、具有特定免疫表型特征的成熟淋巴细胞克隆增殖性肿瘤，以B淋巴细胞在外周血、骨髓、脾脏和淋巴结聚集为特征，CLL以骨髓和外周血受累为主要特征，SLL通常以淋巴结病变为突出表现，二者是同一种疾病的不同表现形式[1]。随着我国老龄化进程的加快，CLL/SLL的发病人数不断上升，给CLL/SLL患者的诊断和治疗带来挑战。

中国抗癌协会血液肿瘤专委会、中华医学会血液学分会、中国临床肿瘤学会（CSCO）、美国国立综合癌症网络（NCCN）、欧洲临床肿瘤学会（ESMO）等权威学术团体发布了一系列CLL/SLL相关诊疗指南[2]–[4]，对CLL/SLL的规范化诊疗提供了重要支撑。然而，不同患者的预后呈高度异质性，复发、难治、高危患者预后较差，治疗难度较大。因此，了解目前临床医生对于CLL/SLL的疾病认知和诊疗情况意义重大。因此，中国抗癌协会血液肿瘤专委会开展了一项全国多中心调查研究，调研对象为全国各地区血液病专科、肿瘤专科与综合医院的各级职称的临床医生，调研重点是阐明目前医生对CLL/SLL的疾病认知和诊疗现况，并通过对比国内外相关文献及数据，发现其与标准治疗间的差距和问题，为进一步推进CLL/SLL的规范诊疗提供依据。

## 病例与方法

一、研究设计

本研究为一项多中心问卷调查研究。研究周期为2021年3月至2021年7月。本研究采用调查员面对面访谈和线上电子问卷调查相结合的方式，共纳入1 000份符合研究要求的调查问卷（线下问卷100份、网络问卷900份）。

二、调查问卷

在中国抗癌协会血液肿瘤专业委员会、中国慢性淋巴细胞白血病工作组的指导下，经过多轮次专家论证，最终形成了涵盖CLL/SLL患者构成、预后评估、高危因素筛查、伴随疾病、脏器功能评估、治疗选择以及布鲁顿酪氨酸激酶（BTK）抑制剂的认知和未满足需求等方面内容的标准问卷。

三、数据处理和分析

所有收集的问卷经过数据核查和处理，排除掉不符合研究要求的数据，最终形成研究分析数据库。本研究的目标为描述医生对CLL/SLL疾病认知和诊疗现况，不涉及统计学分析，主要以对调查收集数据的定量和定性描述分析为主，采用的评价指标主要为平均值、百分比等。

## 结果

一、受访临床医生的基本特征

本研究纳入的1000份调查问卷涵盖全国30个省（直辖市/自治区）中的647家医院，其中48家为CLL中心医院（慢性淋巴细胞白血病工作组参与单位），405家医院为三甲医院，其余医院为三级医院或二级医院。65.2％的受访医生来自血液科或血液肿瘤科，其余34.8％来自肿瘤内科。主任医师、副主任医师和资深主治医师分别占20.6％、57.1％和22.3％。

二、CLL/SLL患者基本情况

受访医生中，年均接诊CLL/SLL患者为33例（CLL 21例，SLL 12例）。CLL中心医院受访医生接诊的年均患者数量（47例）高于其他三甲医院（33例）和非三甲医院（24例），详见表1。在受访医生接诊的CLL/SLL患者中，男性比例（62.0％）高于女性（38.0％），年龄61~70岁的占比最高（37.7％）。CLL、SLL患者的初诊比例分别为43.7％、41.4％，详见表2。在诊断分期方面，初治患者以中危为主（Rai分期Ⅰ/Ⅱ期占47.7％；Binet分期B期占38.0％），复发/难治患者以高危为主（Rai分期Ⅲ/Ⅳ期占61.2％，Binet分期C期占49.5％），详见表3。

**表1:** 各类医院受访医生年均接诊慢性淋巴细胞白血病（CLL）/小淋巴细胞淋巴瘤（SLL）患者数量（例）

组别	CLL	SLL
CLL中心医院	31	16
三甲医院	21	12
非三甲医院	15	9

**注** CLL中心医院：慢性淋巴细胞白血病工作组参与单位

**表2:** 受访医生诊治的慢性淋巴细胞白血病（CLL）/小淋巴细胞淋巴瘤（SLL）患者年龄分布

组别	患者［例（％）］
≤50岁	5 852（14.4）
51~60岁	10 240（25.20）
61~70岁	15 323（37.70）
71~80岁	7 399（18.20）
>80岁	1 830（4.50）

**表3:** 受访医生收治慢性淋巴细胞白血病（CLL）/小淋巴细胞淋巴瘤（SLL）初治、复发/难治患者的临床分期［例（％）］

组别	患者例数	Rai分期			Binet分期		
		0期（低危）	Ⅰ/Ⅱ期（中危）	Ⅲ/Ⅳ期（高危）	A期	B期	C期
初治	15 100	15.4％	47.7％	36.9％	31.1％	38.0％	31.9％
复发/难治	13 485	5.3％	33.5％	61.2％	14.2％	36.3％	49.5％

三、CLL/SLL患者的诊断情况

1. 诊断方法：受访医生采用的诊断方法包括外周血涂片（82.5％）、骨髓穿刺涂片（82.2％）、骨髓活检/免疫组化（68.5％）。

2. 淋巴结活检：80.5％的受访医生选择在淋巴结≥5 cm时开展，而17.6％的受访医生选择在淋巴结直径≥10 cm时开展，有1.9％的受访医生未进行过淋巴结活检。

3. 鉴别诊断：大部分受访医生都认为CLL/SLL需要和套细胞淋巴瘤（MCL，97.1％）、脾边缘区淋巴瘤（SMZL，84.1％）和华氏巨球蛋白血症（WM，65.1％）进行鉴别。

4. 治疗指征：13.7％的受访医生完全掌握了现有指南推荐的起始治疗指征。在CLL中心医院、三甲医院、非三甲医院中，完全掌握现有指南推荐起始治疗指征的受访医生占比为22.4％、13.5％、7.7％。在未掌握治疗指征的受访医生中，48.3％的医生误认为“外周血淋巴细胞计数>100×10^9^/L”是起始治疗指征，61.8％的医生会误认为“有17p−或TP53突变”是起始治疗指征。详见表4。

**表4:** 受访医生对于慢性淋巴细胞白血病（CLL）/小淋巴细胞淋巴瘤（SLL）的起始治疗指征选择情况

起始治疗指征	医生数量及占比［名（%）］
巨块型、进行性或有症状的淋巴结肿大	984（98.4）
进行性骨髓衰竭的证据	916（91.6）
自身免疫性溶血性贫血（AIHA）和（或）免疫性血小板减少症（ITP），对糖皮质激素治疗反应不佳	885（88.5）
进行性淋巴细胞增多	869（86.9）
巨脾（左肋缘下>6 cm）、进行性脾肿大、有症状的脾肿大	864（86.4）
至少存在下列一种疾病相关症状：①在以前6个月内无明显原因的体重下降≥10%；②严重疲乏；③无感染证据，体温>38.0 °C持续≥2周；④无感染证据，夜间盗汗>1个月	862（86.2）
外周血淋巴细胞计数显著升高	827（82.7）
临床试验（符合所参加临床试验的入组条件）	719（71.9）
有del（17p）或TP53突变	618（61.8）
外周血淋巴细胞计数>100×10^9^/L	483（48.3）

四、CLL/SLL患者的预后评估情况

1. CLL国际预后指数（CLL-IPI）评分系统：CLL-IPI评分系统纳入TP53异常（缺失/突变）、免疫球蛋白重链可变区基因（IGHV）突变状态、β_2_-微球蛋白（β_2_-MG）、临床分期、年龄为预后危险分层因素，能够有效地将患者根据预后进行分层，对患者的治疗及预后判断具有重要意义。仅有17.1％的受访医生完全掌握了CLL-IPI的评分标准。在回答错误者中，主要的错误纳入参数包括ECOG评分（63.9％）、11q−（49.1％）和染色体核型（47.6％）。

2. FISH组合检查：FISH检测能够提高相关标志物的检出率，有助于确定预后或治疗决策。在FISH检测项目上，17p−是最常检测的项目（98.6％），其次是13q−（87.1％）、11q−（84.3％）、IgH易位（79.9％）、+12（67.3％）、6q−（51.0％）。

3. 基因检测：在受访医生所接诊的患者中，TP53突变的检测率为67.2％，IGHV基因检测率为63.3％，染色体核型检测率为59.2％。基因检测方式以二代测序（NGS）为主（81.8％）。

4. CLL/SLL预后高危因素：受访医生对于TP53突变（99.3％）、伴复杂核型（84.9％）认知程度较高，对于IGHV未突变（79.7％）、11q−（64.9％）认知程度整体较差。不同等级医院对于危险因素的认知有一定差异，其中CLL中心医院医生的掌握程度要优于三甲医院和非三甲医院。详见表5。

**表5:** 不同等级医院的受访医生对于慢性淋巴细胞白血病（CLL）/小淋巴细胞淋巴瘤（SLL）预后高危因素的认知（％）

组别	受访医生（名）	TP53突变	伴复杂核型	IGHV未突变	del(11q)
CLL中心医院	152	98.7	90.8	88.2	73.7
三甲医院	654	99.4	85.8	78.6	65.7
非三甲医院	194	99.5	77.3	76.8	55.2

五、CLL/SLL患者的脏器功能评估及相关检查情况

在本次调查中，受访医生接诊的CLL/SLL患者主要的伴随疾病包括高血压（31.0％）、感染相关疾病（24.5％）、冠心病（23.5％）、免疫相关疾病（18.4％）、心率失常（14.9％）与其他肿瘤（10.5％）。考虑到伴随疾病，在使用BTK抑制剂治疗时，房颤是医生最常关注的不良反应，对于房颤、肺部感染、出血、胃肠道反应、皮疹、重度骨髓抑制、疼痛和第二原发肿瘤的关注度依次为77.8％、66.1％、57.5％、54.5％、54.1％、43.2％、26.4％和8.6％。由于CLL/SLL为惰性淋巴瘤，大部分患者选择在门诊进行治疗与随访，而门诊患者接受心电图、心脏超声检查的比例分别只有72.7％和55.2％，低于住院患者的95.4％和83.1％。

六、CLL/SLL患者的治疗方案

1. 一线治疗方案：临床上根据患者年龄与高危因素（包括伴17p−/ TP53突变、伴复杂核型、伴IGHV未突变、伴11q−）对治疗方案进行分层。在受访医生过去一年接诊的CLL/SLL患者中，如患者年龄>65岁（无相关高危因素）或存在某一项高危因素，受访医生首选含BTK抑制剂方案，其次为含利妥昔单抗方案；对于年龄≤65岁且无相关危险因素的患者，选含利妥昔单抗方案和选含BTK抑制剂方案作为一线治疗方案的比例接近。从医院类型看，BTK抑制剂的一线使用率在CLL中心医院为49.9％，在三甲医院、非三甲医院分别为38.7％、24.6％。详见表6。

**表6:** 受访医生对不同类型慢性淋巴细胞白血病（CLL）/小淋巴细胞淋巴瘤（SLL）患者的一线治疗选择（％）

组别	BTK抑制剂	利妥昔单抗	苯丁酸氮芥片	化疗	BCL-2抑制剂	临床试验
伴TP53突变	40.7	26.8	10.0	11.8	6.8	4.2
伴复杂核型	40.7	27.7	9.5	11.3	6.8	4.0
伴IGHV未突变	39.6	27.5	10.7	11.7	6.5	3.9
伴del(11q)	39.4	27.1	11.1	12.3	6.4	3.6
年龄>65岁（无相关高危因素）	36.9	25.6	16.0	12.0	6.2	3.3
年龄≤65岁（无相关高危因素）	31.6	32.4	12.4	14.0	6.2	3.5

2. 复发/难治患者的治疗方案：对于复发/难治的CLL/SLL患者，不论患者年龄和高危因素伴随情况，受访医生均首选含BTK抑制剂方案，其次为含利妥昔单抗方案（表7）。在治疗复发/难治患者中，CLL中心医院、三甲医院、非三甲医院受访医生对BTK抑制剂的使用率分别为53％、45％、33％。

**表7:** 受访医生对不同类型复发/难治慢性淋巴细胞白血病（CLL）/小淋巴细胞淋巴瘤（SLL）患者的治疗方案选择（％）

组别	BTK抑制剂	利妥昔单抗	苯丁酸氮芥片	化疗	BCL-2抑制剂	临床试验
伴TP53突变	45.3	23.1	7.9	10.3	8.3	5.1
伴复杂核型	46.0	23.7	7.5	9.7	8.3	4.8
伴IGHV未突变	45.2	23.9	7.9	10.2	8.2	4.6
伴del(11q)	45.4	23.7	8.2	9.9	8.1	4.5
年龄>65岁（无相关高危因素）	43.9	22.4	11.0	10.5	7.9	4.3
年龄≤65岁（无相关高危因素）	39.9	26.7	9.3	11.4	7.8	5.0

七、BTK抑制剂的临床应用情况评价

1. BTK抑制剂疗效优势：相比于化疗，60.9％的受访医生认为BTK抑制剂的优势主要体现在更高的客观缓解率（ORR），53.0％的受访医生认为BTK抑制剂延长高危患者的总生存（OS）时间，41.7％的受访医生认为BTK抑制剂延长了高危患者无进展生存时间（PFS），详见表8。

**表8:** 受访医生认为BTK抑制剂治疗复发/难治慢性淋巴细胞白血病（CLL）/小淋巴细胞淋巴瘤（SLL）相比化疗的主要优势

项目	受访医生［名（%）］
更高的客观缓解率	690（60.9）
延长高危患者的总生存时间	530（53.0）
延长高危患者的无进展生存时间	417（41.7）
更长的缓解持续时间	390（39.0）
更高的完全缓解率	283（28.3）
延长所有患者的总生存时间	214（21.4）
更低的不良事件发生率	175（17.5）
延长所有患者的无进展生存时间	152（15.2）

2. BTK抑制剂的减量使用和中断治疗：78.6％的受访医生认为减量使用BTK抑制剂会影响疗效，但在实际临床治疗中仍有69.0％的医生曾减量使用过BTK抑制剂。受访医生表示，减量使用BTK抑制剂的主要原因是不良反应（100.0％）、患者经济因素（50.0％）以及疗效得到控制（20.0％）。虽然56.1％的受访医生认为中断BTK抑制剂会影响疗效，但在实际临床治疗中仍有66.8％的医生曾中断BTK抑制剂。受访医生认为平均中断17.0 d会影响疗效，但在临床平均中断时间为12.1 d，远高于相关研究报道中的7 d[5]。

3. BTK抑制剂的终止治疗：对于初治患者，受访医生终止BTK抑制剂治疗的原因包括疾病进展（35.0％）、不良反应（32.8％）和经济因素（32.1％）；对于复发/难治患者，BTK抑制剂终止治疗的原因包括疾病进展（42.5％）、不良反应（30.3％）和经济因素（27.2％）。大部分医生表示，当患者出现房颤、重度骨髓抑制、出血、肺部感染、第二原发肿瘤、胃肠道反应、皮疹和疼痛时，他们会选择中断或终止BTK抑制剂的治疗，中断/终止率依次为70.6％、63.7％、52.3％、49.0％、15.9％、7.5％、7.4％和3.5％。

八、血液科和肿瘤科医生在CLL/SLL诊疗中的差异

1. CLL/SLL预后评估差异：在CLL-IPI评分系统方面，20.9％的血液科医生完全掌握了CLL-IPI评分标准，而掌握该评分标准的肿瘤科医生占比为11.3％。

2. 治疗方案差异：在一线治疗中，无论患者是否有高危因素或年龄是否>65岁，BTK抑制剂方案均是血液科和肿瘤科医生最常用的一线治疗选择，且血液科医生在各人群中的BTK抑制剂使用比例普遍高于肿瘤科医生。详见表9。

**表9:** 血液科与肿瘤科受访医生对慢性淋巴细胞白血病（CLL）/小淋巴细胞淋巴瘤患者的治疗选择（％）

科室	BTK抑制剂	利妥昔单抗	苯丁酸氮芥片	化疗	BCL-2抑制剂	临床试验
血液科医生						
一线治疗	42.7	27.5	10.6	10.3	5.7	3.2
复发/难治	49.3	23.1	7.0	8.6	7.7	4.3
肿瘤科医生						
一线治疗	28.7	28.5	13.9	16.0	8.0	4.9
复发/难治	34.0	25.5	12.0	13.9	8.8	5.7

3. 受访医生对BTK抑制剂减量的认知和实践：84.4％的受访血液科医生和60.0％的受访肿瘤科医生认为减量使用BTK抑制剂会影响疗效；在实际临床治疗中，62.5％的受访血液科医生和90.0％的受访肿瘤科医生曾减量使用过BTK抑制剂。

## 讨论

本研究通过对全国30个省（直辖市/自治区）647家各级医疗机构的1 000名血液科、肿瘤科或淋巴瘤科临床医生进行问卷调研，对各级医师对于CLL/SLL患者诊断、预后评估、治疗的现状进行了解。研究结果将对后续进一步规范化CLL/SLL的诊疗工作提供重要依据。

本研究显示，受访医生年均接诊的CLL/SLL患者数量较少，且患者的诊疗集中度不高。就本研究结果来看，中国CLL/SLL患者较美国更年轻，81.1％的中国CLL/SLL患者位于51~80岁年龄区间，而79.9％的美国CLL/SLL患者年龄≥60岁，其中超过80岁的患者占比为24.1％[6]。尽管目前中国CLL/SLL患者的人流行病学特征尚未见公开发表数据，本研究的数据还是可以为初步了解CLL/SLL患者的发病特征提供一些参考。

血常规和外周血涂片在CLL/SLL的疾病诊断中是最基础的检查项目[3]。在诊断CLL/SLL时，82.5％的受访医生会进行外周血涂片，82.2％的医生进行了骨髓涂片和骨髓活检。虽然CLL通过外周血就能诊断，但68.5％以上的患者需要进行鉴别，骨髓穿刺活检是与其他B淋巴细胞增殖性疾病鉴别的重要手段，尤其是与MCL、SMZL和WM进行鉴别诊断[7]。此外，虽然淋巴结活检对CLL患者无诊断作用，但是当患者淋巴结直径≥5 cm需高度警惕Richter综合征转化和组织学进展。Rossi等[8]也在研究中指出，淋巴结直径≥5 cm时，应行活检以排除Richter转化。

本次调研结果显示，32.7％的患者初诊时无治疗指征，这一结果与流行病学数据[9]基本吻合。然而，本调研中只有13.7％的受访医生能够正确掌握CLL/SLL患者的治疗指征。因此，CLL/SLL的治疗指征亟待向全国各级医院的医生进行推广。

目前最常采取Rai和Binet两种临床分期系统判断患者的预后，这两个系统仅依赖体检和简单实验室检查，具有一定的局限性[10]–[11]。目前随着研究的不断深入，IGHV基因无突变、合并17p−和（或）TP53基因突变、11q−在不同研究中均提示与CLL不良预后相关[12]；染色体复杂核型异常、NOTCH1突变、SF3B1突变、BIRC3突变、MYD88突变、CD38、ZAP70及CD49d高表达等均与CLL不良预后相关[13]–[16]。CLL-IPI评分系统纳入了TP53缺失和（或）突变、IGHV基因突变状态、β_2_-MG、临床分期、年龄为预后危险分层因素[17]，在中国CLL患者中具有较好的预后评估价值[18]–[19]。本研究结果提示，82.9％的受访医生不能正确使用CLL-IPI评分系统，在实际临床中也未能对患者进行CLL-IPI评分，进而导致CLL/SLL患者的预后风险未能被准确识别，这将不利于患者长期管理。此外，在CLL/SLL高危因素的认知上，受访医生对于IGHV未突变和11q−这两个预后高危因素的掌握情况较差，而这两群患者是BTK抑制剂的获益人群。因此，进一步提升医生对IGHV未突变和11q−等CLL/SLL预后高危因素的认知非常关键。

CLL患者发病年龄较大，基础疾病较多，常伴有心血管疾病等并发症，在接受BTK抑制剂治疗时，更易发生房颤等不良事件；且由于免疫功能减退容易引发感染，许多CLL患者的最终死亡原因是与血液系统疾病无关的其他疾病。因此，应向医生强调伴随疾病评估对于患者治疗的重要影响，鼓励更多的患者在治疗前和治疗过程中仔细排查、留心观察基础疾病或伴随疾病从而降低患者因伴随疾病造成死亡的风险和概率。随着BTK抑制剂等靶向治疗的可及性提高，更多的CLL/SLL患者主要在门诊进行治疗，然而本研究发现门诊患者的心脏相关功能检测、病毒检测、免疫相关功能检测存在不足。因此在推广和加强伴随疾病评估时，尤其要强调针对门诊患者的评估，提升医生对于心血管评估的重视，可以通过拟定CLL/SLL伴随疾病评估管理指南等方式，逐步建立起以患者为中心、多学科、全周期的规范化治疗评估体系，为各级医生的疾病筛查与患者管理提供参考。

目前，CLL仍为一种难以治愈的疾病。最早期是使用烷化剂、嘌呤类似物作为主要的治疗手段。随着CD20单克隆抗体的出现，开始了以利妥昔单抗为基础的免疫化疗时代。目前，已经进入了BTK抑制剂为主的小分子靶向治疗时代。中国CLL指南对于有治疗指征的CLL/SLL患者根据不同的风险分层（基于基因突变、伴随疾病）推荐了一系列治疗方案[4]。

多项临床试验显示，BTK抑制剂相比免疫化疗能够显著改善初治和复发/难治CLL/SLL患者的长期生存，尤其能显著改善伴高危因素患者的生存且不良反应可控[20]。在BTK抑制剂的选择上，ALPINE研究对伊布替尼和泽布替尼在复发/难治CLL/SLL患者中进行头对头评价[21]，中期结果提示泽布替尼的治疗有效率（ORR）及12个月PFS率均显著优于伊布替尼[22]。本研究调查显示，尽管受访医生在一线治疗和复发/难治患者中应用BTK抑制剂的比例较高，但与指南建议相比，受访医生对于BTK抑制剂的优势患者人群认知仍不够清晰，且减量用药和中断用药的发生率比预期要高。而疾病进展、不良反应和经济因素是医生选择终止BTK抑制剂的主要原因，主要不良反应是房颤、重度骨髓抑制、出血以及肺部感染等。有研究显示BTK抑制剂中断超过7 d或减量使用会对患者的长期预后产生显著影响[23]。因此，需要对医生进一步加强BTK抑制剂规范使用和不良反应管理，减少治疗中断时间以及减量服用的情况发生。

血液科是收治CLL/SLL患者的主要科室，血液科医生相关诊疗经验相对更丰富，而肿瘤科医生接诊CLL/SLL患者的数量比较少，因此，对于CLL/SLL疾病的了解、预后高危因素的认知、治疗方案的选择、BTK抑制剂的临床应用和不良反应管理等方面需要进一步加强。

综上所述，本研究结果显示目前我国医生在CLL/SLL的规范化诊疗方面仍有很多不足，需要从不同层面、多维度努力提高我国各级医生对本病的认知，从而进一步提高我国CLL/SLL诊疗水平。
